# Forced Oscillation Technique for Monitoring the Respiratory Status of Children with Cystic Fibrosis: A Systematic Review

**DOI:** 10.3390/children8100857

**Published:** 2021-09-27

**Authors:** Ioanna Loukou, Maria Moustaki, Agni Deligianni, Olympia Sardeli, Konstantinos Douros

**Affiliations:** 1 Cystic Fibrosis Department, “Agia Sofia” Children’s Hospital, 11527 Athens, Greece; mar.moustaki@gmail.com (M.M.); agnideli@gmail.com (A.D.); 2 Pediatric Allergy and Respiratory Unit, 3rd Department of Pediatrics, “Attikon” University Hospital, School of Medicine, National and Kapodistrian University of Athens, 12462 Athens, Greece; ol.sardeli@googlemail.com

**Keywords:** cystic fibrosis, forced oscillation, oscillometry, spirometry

## Abstract

Spirometry is considered the gold standard method for monitoring lung function of patients with cystic fibrosis (CF) but it requires patients’ cooperation and therefore it is not useful for the majority of preschool-aged children. Oscillometry is an alternative modality for lung function monitoring that requires minimal cooperation and can be applied in children as young as 3 years of age. Furthermore, it generates lesser aerosol compared to spirometry, an issue that is of considerable importance in the COVID-19 era. The aim of this review was to present the existing clinical data regarding the application of oscillometry in children and adolescents with CF. The method seems to have acceptable feasibility and repeatability. However, there is conflicting data regarding the correlation of oscillometry values with the clinical symptoms of CF patients either in clinically stable or in exacerbation periods. Furthermore, it is not clear to what extent oscillometry measurements correlate with the spirometry indices. Based on current evidence, spirometry cannot be substituted by oscillometry in the monitoring of the respiratory status of children and adolescents with CF.

## 1. Introduction

Cystic fibrosis (CF) is a multi-system genetic disease with a considerable burden of morbidity that is mainly attributed to respiratory health deterioration. Mortality is not negligible and is attributed mainly to lung disease progression and respiratory complications. Therefore, monitoring of respiratory health of patients with CF, during both clinical stability and respiratory exacerbations, is mandatory. Spirometry is the most commonly applied method for monitoring lung function of school-aged children, adolescents, and adults with CF [1,2]. An inherent limitation of spirometry is that it requires a patient’s cooperation and therefore is usually not applicable in children younger than 6 years old. Therefore, other modalities of lung function assessment such as forced oscillation technique [3] and breath washout [4] have been explored in preschool-aged children as well as in older children and adolescents.

Forced oscillation technique (FOT) requires minimal cooperation from the patient as it is effort-independent. All it needs is resting tidal breathing through a mouthpiece while sound waves of different frequencies are superimposed through a loudspeaker. It has been used mainly for monitoring patients with asthma but is increasingly being applied in patients with CF. It is of note that in the era of the COVID-19 pandemic, there is a growing interest in oscillometry as it is a procedure with lesser aerosol production than spirometry [5].

However, for the time being, it is not possible to replace spirometry with oscillometry as its clinical utility in CF monitoring is supported only by a small number of studies with variable and rather inconsistent results. Indeed, more large and well-designed studies are needed to fully assess the potential of oscillomerty in the longitudinal evaluation of CF disease progression.

The aim of this systematic review was to critically present the existing data regarding the application of oscillometry and describe its strengths and limitations in children and adolescents with CF.

## 2. Methods

**Figure 1:**
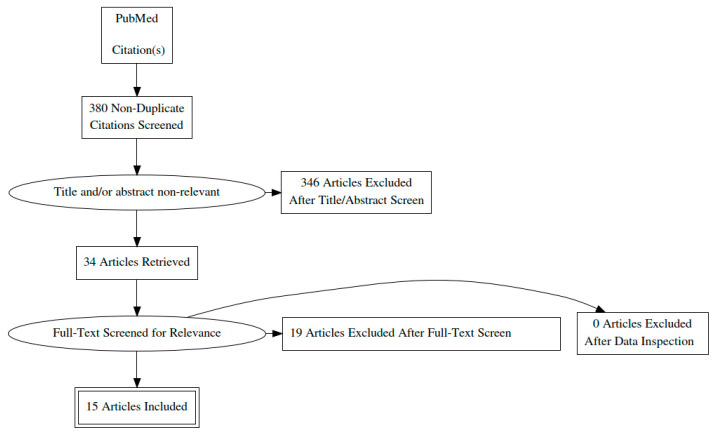
PRISMA diagram of the literature search process.

The PRISMA guidelines were used to structure this review. We performed a search in the PubMed database using the following algorithm: (((oscillometry) OR (oscillation)) OR (impedance)) AND (cystic fibrosis). The search retrieved 380 publications. The articles were eligible for review if they were written in English and included patients less than 18 years old. There was no chronological limitation. During the title and abstract screening, 346 articles were excluded as being not relevant to the subject, referring to patients older than 18 years, written in a non-English language, or being reviews. In addition, 19 more studies were excluded after reading the full texts. Finally, 15 articles were included in this systematic review. The literature search process is summarized in the form of a PRISMA diagram in Figure 1.

Given that the final number of the studies was small, we decided to include all of them in this systematic review without performing a quality assessment. Furthermore, the studies were included irrespective of the type of device and the frequency values that were used in each one. This approach was chosen—despite its obvious limitations—in order to provide the reader with an overview of the (rather scarce) literature in this area. A summary of the studies that were included in this review is shown in Table A1 (Appendix A).

## 3. Result

### 3.1. Forced Oscillation Technique-Background

FOT was initially described by Dubois in 1956 [6]. Since then, several FOT variants were developed regarding measurement patterns, oscillation frequencies and assessment principles. More recently, the impulse oscillometry technique (IOS) was developed, which generates pressure oscillations at a standard square pressure wave, at a frequency of 5 Hz from which all other applied frequencies are derived using spectral analysis.

FOT measures the airway impedance (denoted as Zrs) which is comprised of two components: the real part, which is the resistance (denoted as Rrs), and the imaginary part, which is the reactance (denoted as Xrs). The resistance is the in-phase component of respiratory impedance which reflects the relationship between the applied pressure and the resultant flow. The reactance is the out-of phase component of impedance and encompasses the capacitive and inertive properties of the lung [3,7].

The resistance represents the total respiratory system resistive properties (extrathoracic and intrathoracic airways, lung parenchyma, and chest wall). The resistance at low frequencies (i.e., 5 Hz) reflects the total airway resistance while the resistance at high frequencies (i.e., 20 Hz) reflects large airway resistance, as high-frequency sounds do not reach the small airways. The difference between resistance at 5 Hz and resistance at 20 Hz reflects the small airway resistance.

The reactance comprises the inertia of the air column to move which is positive in sign, and the capacitance of the lung which reflects the elasticity of the lung and is negative in sign. For both technical and physiological reasons oscillometry results are most often reported as the resistance and reactance at 5 Hz (Rrs5 and Xrs5, respectively), and the resistance and reactance at 20 Hz (Rrs20, Xrs20, respectively), although the results from other frequencies may also be reported.

The frequency at which the reactance crosses zero is called resonant frequency (denoted as Fres). At this point, the capacitive properties are equal and opposite to the inertive forces. Below this frequency capacitive properties are dominant whereas above this frequency inertive properties dominate [7].

Another important measurement of oscillometry is AX which represents the sum of all reactance components at all frequencies before the resonant frequency. It reflects the total area that capacitance is dominant reflecting the elastic properties of the lung [8].

For the performance of the FOT procedure, the children should be sitting calm, containing their cheeks into their hands, and not inserting their tongue into the mouthpiece [9]. The subjects should breathe quietly at the level of tidal breathing and three to five consecutive breathes should be analyzed with a coefficient of variation less than 15% for children [7].

### 3.2. Feasibility and Reliability

In healthy children, the oscillation technique is feasible even from the age of three years, as there is no need for an expiratory maneuver, as in spirometry. The within-test variation, as it was demonstrated by Hall et al. in healthy children, is satisfactory; it is higher in children who have their serial attempts without any rest in between [10]. Concerning the short-term repeatability within 15 min, the relative mean difference was 2% for all FOT variables except for reactance, which showed a relative mean difference of 3.8%.

The feasibility was also assessed in children with CF. Kerby et al. evaluated spirometry and forced oscillation in children with CF and healthy controls aged 36 to 60 months [11]. Acceptability rates did not differ between patients and controls, and between males and females, for each age range. They were higher compared to those of spirometry. The overall acceptability rate was 70% for FOT among children with CF and 72% among healthy controls. However, for children with CF aged between three and four years, the acceptability rate was as low as 40%. The intraclass correlation coefficient (ICC) for two-week interval reproducibility was moderate for resistance and reactance but lower to the respective ICC for spirometric measurements at the same interval. Higher ICC was found in another study [12] in children with CF and healthy controls; however, the age range was 6–14 years, and therefore, this difference could be attributed to the different age range from the study of Kerby et al. [11]

In another study [13] in children aged 2–7 years with CF, it was shown that the between-test repeatability within a 15 min interval was acceptable and the observed difference was <3% for resistance and <13% for reactance. Compared with the results obtained by Hall et al. in healthy children [10], the values in CF children were similar for the resistance and higher for the reactance. It is of note that within the CF study population there was no difference for repeatability between those with and without symptoms.

### 3.3. Correlation of Oscillometry and Spirometry Values in Children with Cystic Fibrosis

Spirometry is considered the gold standard method for the assessment of lung function. Therefore, several studies have evaluated the correlation between spirometry and oscillometry indices in children with CF. In 1997 Lebecque et al. [14] investigated this correlation in both asthmatic and CF children. The Rrs was measured at 10 Hz. According to their findings, FEV1 and Rrs showed concordant results in only 16/45 studied children with CF. They also found that Rrs failed to detect even severe airway obstruction as this was indicated by FEV1. The results were not surprising as CF affects mainly the peripheral airways and this obstruction can be depicted by FEV1 but not by Rrs at 10 Hz (which mainly reflects the large airways resistance). The authors did not use other frequencies nor did they assess reactance or other parameters. Similarly, when different lung function tests were evaluated in children aged 2–8 years with CF, no significant correlation between FEV1 with Xrs and Rrs at 5 Hz was found [15]. A similar study was also conducted later by Moreau et al. [16] in children with CF, aged 4–19 years, that evaluated FOT at the same frequency of 5 Hz. They also did not find any significant correlation between FEV1 and FOT values when the indices were expressed as a percent of predicted values. They only found a significant correlation between raw data. More recently, however, in another study [17] that enrolled 34 children and 5 adults, it was shown that there was a significant negative correlation between FEV1 and Zrs5, Rrs5 and Rrs20 when the indices were expressed either as a percent of the predicted values or as raw data. Xrs5 correlated with FEV1 at a significant level only when raw data were considered. Furthermore, Zannin et al. [18] studied the relationship of FOT values with spirometry. They also studied the within-breath changes in respiratory impedance with spirometric indices. They found that both Rrs8 and expiratory Rrs8 were negatively correlated with FEV1. Reactance at 8 Hz and expiratory Xrs8 were both positively correlated with FEV1. The difference between the inspiratory and expiratory reactance was negatively correlated with FEV1 whereas inspiratory values were not correlated with any lung function parameters. Reactance was more severely affected than resistance, indicating that peripheral airways were more severely obstructed. A most recent retrospective study showed significant correlations between spirometry and many FOT indices with parallel changes after bronchodilator administration [19].

### 3.4. Distinguishing Subjects with Cystic Fibrosis from Healthy Controls by Oscillometry

In 1973 Cogswell [20] used the FOT in healthy children, children with asthma, and children with CF. Although the authors did not compare in their analysis the results of healthy and CF children, it was mentioned that many of the children with CF had Rrs within the normal range, although the values tended to increase with age and during exacerbations. In 1985 Solymar et al. [21] studied, among other patients, 13 children with CF and found that only 4 had abnormal Rrs values; the reactance at 2 Hz was the most discriminative variable, as it was abnormal in 6/13 patients. A similar study in 2011 compared preschoolers with CF and healthy controls of the same age and showed that FOT indices were not different between clinically stable CF patients and healthy controls, whereas acceptable measurements for spirometry were lower for children with CF compared to controls [11].

### 3.5. Oscillometry and Its Association with the Clinical Condition in Subjects with CF

Oscillometry has been evaluated in patients with CF before exacerbation and following a 14-day treatment regimen at the hospital [22]. It was found that there was a significant decrease of absolute values of reactance and resistance at 5 Hz by 22% and 27% respectively and these differences were statistically significant. In contrast, the resistance measured at levels >10 Hz and reactance measured at levels >15 Hz did not show any significant change. There was also a significant increase of FEV1 by 27%. Similarly in 2015 Buchs et al. [23] noticed that there was a significant change of reactance and resistance at 5 Hz by 22% and 13%, respectively, following intravenous antibiotic treatment in patients at exacerbation whilst there was a significant increase of FEV1 by 20%. The higher amplitude of change among FOT variables was observed for Xrs5 and Xrs10. However, when a cutoff value for clinically relevant improvement was considered as 10% for FEV1 and 20% for Xrs5, 79% of the studied patients were classified as having improved based on the FEV1 change, whereas only 44% of them were classified as having improved according to the Xrs5 change criterion. Therefore, the sensitivity of oscillometry for tracking clinical improvement was inferior to that of spirometry. Furthermore, it was noticed that there was a significant correlation between FEV1 and ΔFEV1 but not between Rrs5 and ΔRrs5 as well as between Xrs5 and ΔXrs5. This implied that FEV1 changes were dependent on initial FEV1 values, whereas the same thing did not happen for oscillometry indices.

Oscillometry indices were also evaluated in asymptomatic and symptomatic children with CF in relation to their symptoms [13] and it was found that children with current symptoms have significantly decreased reactance and increased resistance at 6 Hz. Collectively, both symptomatic and asymptomatic children showed increased resistance at 6 Hz, 8 Hz and 10 Hz compared to the healthy reference population and decreased reactance at 6 Hz and 8 Hz but not at 10 Hz. In contrast to these results, Ren et al. [22] did not find any association of FOT measurements with pseudomonas status, cough score, family history of asthma and other variables. No longitudinal association was observed between FOT values and pseudomonas status of children with CF. The authors attributed the discrepancy with the results of Gangell et al. [13] study to the fact that that the studied children were clinically stable. A recent study that evaluated retrospectively FOT values with clinical status found a significant correlation between FOT parameters and clinical symptoms [19].

### 3.6. Oscillometry and Its Association with Pulmonary Inflammation, Infection, and Structural Lung Disease, in Subjects with CF

Another issue that received the attention of researchers was whether FOT measurements were related to the presence of inflammation and infection markers in bronchoalveolar lavage (BAL) fluid. In a study that included a small number of patients aged younger than 6 years, it was shown that FOT was sensitive at detecting early lung disease, as the latter was defined by the presence of inflammation in BAL fluid [24]. Some years later Ramsey et al. [25] found that the BAL neutrophil count of 184 longitudinally studied children was associated with an increase of Fres z-score and Interleukin 8 with a lower reactance z-score at 8 Hz, whereas neutrophil elastase was not associated with any of the FOT values. They also showed that only the isolation of Haemophilus influenza in the BAL was associated with increased resistance and decreased reactance z-scores at 8 Hz. No association was found between other bacterial pathogens and any FOT parameters. The only structural lung disease that was associated longitudinally with a decrease of reactance z-score at 8 Hz was the presence of air trapping. They concluded that FOT parameters were not sensitive enough to detect the underlying lung disease either cross-sectionally or longitudinally. The most sensitive indices were Fres and reactance at 8 Hz. A limitation of this study, however, was that at the time of BAL collection, the studied children were clinically stable and therefore these findings did not provide an insight into the correlation of FOT parameters with the clinical symptoms and disease exacerbations. No relation was found between FOT values and MRI CF morphological scores as evaluated by Zannin et al. [18] indicating that FOT should not be used as a substitute for imaging studies.

## 4. Conclusions

From the above-presented data, it becomes evident that the role of oscillometry as a lung function modality for monitoring respiratory status in children and adolescents has not yet been established. More studies are needed in order to evaluate the longitudinal relationship of patients’ clinical condition and oscillometry values, as well as the relationship of oscillometry indices with those of spirometry, since the existing studies are, in their majority, of small size and their results are conflicting. In the era of the COVID-19 pandemic, there is an increasing interest in oscillometry, as it is a procedure with lesser aerosol generation compared to spirometry. This is another non-negligible reason for clarifying whether oscillometry can provide useful information for the respiratory status of children and adolescents with CF while on exacerbation or clinical stability.

## Abbreviations

CF: Cystic fibrosis; FOT: Forced oscillation technique; IOS: Impulse oscillometry technique; Zrs: airway impedance; Xrs: Reactance; Fres: Resonant frequency; FEV1: Forced expiratory volume in the first second; BAL: Bronchoalveolar lavage.

## Appendix A

**Table A1:** A summary of the studies that were included in this review.

	Author	Year	Main Outcome Measures	Main Recorded Parameters
Studies on feasibility and reliability of FOT in children with CF				
1a	Kerby G	2011	Feasibility and short term reproducibility of oscillometry	Rrs and Xrs at 4, 6, 8 and 10 Hz
2	Wamosy RMG	2021	Assessment within trial reliability of 3 measures in IOS parameters.	Rrs at 5 and 20 Hz, Zr at 5 Hz, Xr at 5 Hz
3a	Gangell C.L	2007	Evaluation of between measurement repeatability of oscillometry	Zrs, Rrs and Xrs at 6, 8 and 10 Hz
Studies on correlation between oscillometry and spirometry values				
4	Lebeque P	1997	Evaluation of Rrs to detect airway obstruction in patients with CF and in patients with bronchial asthma	Rrs at 10 Hz
5	Nielsen K	2004	Assessment of the usefulness of IOS in the evaluation of lung function	Rrs at 5 and 20 Hz, Xrs at 5 Hz, Zrs at 5 Hz
6	Moreau L	2009	Assessment of IOS values to quantify lung function impairment in relation to the respective spirometric indices at a cross sectional and longitudinal level	Rrs, Xrs, Zrs at 5 Hz
7	Raj D	2014	Evaluation of IOS utility for the detection of airway obstruction and its correlation with spirometric indices	Rrs at 5 and 20 Hz, Zrs, XRs at 5 Hz
8	Zannin E	2019	Evaluation of the relationship of FOT parameters with other lung function indices such as spirometry	Zrs at 8 Hz
9a	Ozturk K	2021	Evaluation of the relationship of FOT parameters with spirometry values	Rrs and Xrs at 5, 10 and 20 Hz
Studies on the discrimination of subjects with CF from healthy subjects based on IOS values				
10	Cogswell J.J	1973	The use of oscillation technique in the evaluation of healthy children and in those with obstructive airway disease (asthma or cystic fibrosis)	Forced oscillation applying a sine wave frequency from 5–7 Hz.
11	Solymar L	1985	The use of oscillation technique in the evaluation of healthy children and in those with cricoid stenosis, cystic fibrosis or asthma	Rrs, Xrs and Zrs at 2, 4, 12 Hz
1b	Kerby	2011	Comparison of the ability of the technique to differentiate patients with CF from healthy controls	Rrs and Xrs at 4, 6, 8 and 10 Hz
Studies on the correlation of clinical condition with oscillometry values				
12	Ren C.L	2011	Correlation between clinical features of CF patients with oscillometry indices and other lung function tests	Rrs and Xrs at 4, 6, 8 and 10 Hz
13	Buchs C	2015	Evaluation of oscilometry responsiveness properties in children with CF while on acute exacerbation, prior and following intravenous antibiotic treatment	Rrs and Xrs at 5, 10, 15 and 20 Hz.
3b	Gangell C.L	2007	Oscillometry values in symptomatic and asymptomatic children with CF	Zrs, Rrs and Xrs at 6, 8 and 10 Hz
9b	Ozturk K	2021	Evaluation of the relationship of FOT parameters with clinical scores	Rrs and Xrs at 5, 10 and 20 Hz
Studies on the correlation of oscilometry values with pulmonary inflammation, infection and structural changes				
14	Brennan S	2005	Evaluation of the correlation of inflammation and infection detected in BAL fluid with lung function including oscillometry	Zrs after applying a broadband forcing signal 0.5–20 Hz.
15	Ramsey K	2015	Assessment of the association between inflammation, infection and structural lung disease with oscillometry values	Rrs and Xrs at 8 Hz.
